# Renoprotective Effect of Vardenafil and Avanafil in Contrast-Induced Nephropathy: Emerging Evidence from an Animal Model

**DOI:** 10.3390/jpm12050670

**Published:** 2022-04-22

**Authors:** Ioannis-Erineos Zisis, Georgios Georgiadis, Anca Oana Docea, Daniela Calina, Liliana Cercelaru, John Tsiaoussis, Georgios Lazopoulos, Nikolaos Sofikitis, Aristidis Tsatsakis, Charalampos Mamoulakis

**Affiliations:** 1 Department of Urology, University General Hospital of Heraklion, Medical School, University of Crete, 71003 Heraklion, Crete, Greece; renoszisis@gmail.com (I.-E.Z.); geokosgeo@gmail.com (G.G.); 2 Department of Toxicology, University of Medicine and Pharmacy of Craiova, 200349 Craiova, Romania; 3 Department of Clinical Pharmacy, University of Medicine and Pharmacy of Craiova, 200349 Craiova, Romania; calinadaniela@gmail.com; 4 Anatomy and Embryology, University of Medicine and Pharmacy of Craiova, 200349 Craiova, Romania; cercelaruliliana@gmail.com; 5 Laboratory of Anatomy-Histology-Embryology, Medical School, University of Crete, 71003 Heraklion, Crete, Greece; tsiaoussis@uoc.gr; 6 Department of Cardiac Surgery, University General Hospital of Heraklion, Medical School, University of Crete, 71003 Heraklion, Crete, Greece; g.lazopoulos@uoc.gr; 7 Department of Urology, School of Medicine, Ioannina University, 45110 Ioannina, Epirus, Greece; v.sofikitis@hotmail.com; 8 Department of Forensic Sciences and Toxicology, Medical School, University of Crete, 71003 Heraklion, Crete, Greece; tsatsaka@uoc.gr

**Keywords:** acute kidney injury, contrast media, avanafil, vardenafil, phosphodiesterase 5 inhibitors, nephropathy, kidney

## Abstract

The potential renoprotective effects of vardenafil (VAR) have been evaluated in a very limited number of studies using acute kidney injury animal models other than contrast-induced nephropathy (CIN) with promising results, while avanafil (AVA) has not been evaluated in this respect before. The purpose of this study was to evaluate for the first time the potential renoprotective effect of VAR and AVA in a rat model of CIN. Twenty-five male Wistar rats were equally assigned into five groups: control, CIN, CIN+N-acetyl cysteine (NAC) (100 mg/kg/day) as a positive control, CIN+VAR (10 mg/kg/day) and CIN+AVA (50 mg/kg/day). CIN was induced by dehydration, inhibition of prostaglandin and nitric oxide synthesis as well as exposure to the contrast medium (CM). Serum Cr (sCr) levels were measured at 24 and 48 h after CIN induction. At 48 h of CM exposure, animals were sacrificed. Matrix metalloproteinase (MMP) 2 (MMP-2) and MMP-9, kidney injury molecule 1 (KIM-1) and cystatin-C (Cys-C) were measured on renal tissue. Histopathological findings were evaluated on kidney tissue. All treatment groups had close to normal kidney appearance. sCr levels subsided in all treatment groups compared to CIN group at 48 h following CIN induction. A significant decline in the levels of MMP-2, MMP-9, KIM-1 and Cys-C compared to CIN group was observed. These results provide emerging evidence that VAR and AVA may have the potential to prevent CIN.

## 1. Introduction

Contrast-induced nephropathy (CIN) is a serious iatrogenic form of acute kidney injury (AKI) that occurs 24–72 h after exposure to iodinated contrast media (CM) [1,2]. The pathophysiology of CIN remains obscure, but oxidative stress appears to play a key role [3,4]. Detection of novel biomarkers assisting in the elucidation of pathophysiology, accurate diagnosis, and follow-up of affected individuals are currently attracting considerable attention [5]. CIN management remains supportive based mainly on intravenous preventive hydration recommended in patients at-risk, with either saline or bicarbonate protocols showing similar efficacy [6,7].

Several antioxidants have also been investigated but none of them, including N-acetyl cysteine (NAC) (the most widely investigated medication), has been proven to be effective in this respect in human studies to date [6,7]. Nevertheless, recent systematic reviews continue to report that NAC shows promise for preventing CIN [8] and a 2019 National Institute for Health and Care Excellence review recommended that more clinical research be performed [9]. As a result, NAC is still commonly used in daily clinical practice as previously described [10]. Last but not least, promising results have been reported in several animal studies using NAC as either the main treatment under investigation or standard/conventional treatment (positive control) [11,12,13,14,15,16,17,18,19,20,21,22]. Apart from its antioxidant properties [23], supporting biological plausibility of efficacy relies on its vasodilatory effects [24], namely its ability to cause renal artery vasodilatation/renal blood flow increase, improving renal hemodynamics [10].

**Figure 1:**
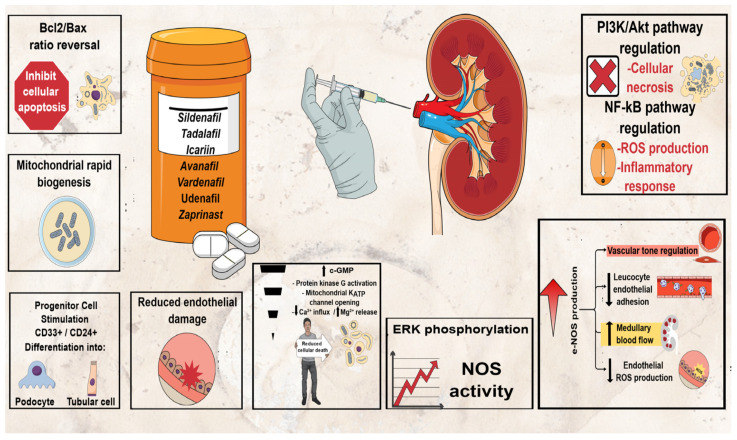
Potential renoprotective mechanisms of PDE5Is in AKI.

In these terms, the potential renoprotective effects of phosphodiesterase 5 inhibitors (PDE5Is), currently approved for treating erectile dysfunction (four selective PDE5Is: sildenafil, tadalafil, vardenafil (VAR) and avanafil (AVA)); pulmonary arterial hypertension (sildenafil and tadalafil); and lower urinary tract symptoms secondary to benign prostatic obstruction (tadalafil) [25,26,27], have also been tested in a number of animal studies showing promising results for preventing AKI [28]. Consequently, the beneficial effect of PDE5Is in preventing AKI represents one of their emerging therapeutic applications among many others reported [27,28,29,30,31,32,33]. The mechanism of action of PDE5Is in AKI prevention/management is still obscure. Multiple mechanisms have been proposed to play a role in counteracting the cascade of changes caused by renal injury, the most common of which are summarized in Figure 1 [28]. These medications have shown beneficial potential through various mechanisms in a few CIN animal models in particular, but such data are scarce, limited to sildenafil and tadalafil to the best of our knowledge [3,28]. The potential renoprotective effects of VAR have been evaluated in a very limited number of studies using AKI animal models other than CIN with promising results, while AVA has not been evaluated in this respect before [28]. The aim of the present study was to evaluate for the first time the potential renoprotective effects of VAR and AVA in a CIN rat model.

## 2. Materials and Methods

### 2.1. Animals

Twenty-five Wistar male rats, 6 months old, weighting 300–350 g were acquired from the University of Medicine and Pharmacy Craiova Animal House. The rats used were 6 months old which corresponds to humans of 18 years old, corresponding to young healthy adults so no intervention of the aging is possible in the CIN induction [34]. All rats were housed and acclimatized for 14 days (constant temperature 21 ± 2 °C, 12 h/12 h dark/light cycle) prior to the initiation of the experiment. All animals were provided food and water ad libitum. Prerequisites for the experimental process were in accordance with the EU Commission Directive 2010/63/EU. The experimental protocol was reviewed and authorized by the Ethical Committee of the University of Medicine and Pharmacy of Craiova, Romania.

### 2.2. Experimental Design

After acclimatization, all animals were divided into 5 equal groups (*n* = 5 animals in each group) and treated for 7 days as follows: The duration of treatment for VAR/AVA and NAC was based on the results obtained by our group for sildenafil and tadalafil [22] and on a previous study evaluating the renoprotective effect of NAC [14], respectively. The 1st group (Control group) received 0.5 mL corn oil once per day by oral gavage. The 2nd group (CIN group) received 0.5 mL corn oil once per day by oral gavage before CIN induction. The 3rd group received *N*-acetyl cysteine (NAC group) (Sigma-Aldrich, St. Louis, MO, USA) 100 mg/kg once per day by oral gavage before CIN induction. The dose of NAC was selected based on a previous study evaluating the renoprotective effect of NAC [14]. The 4th group received VAR (VAR group; ≥98% (high-performance liquid chromatography (HPLC))) (Sigma-Aldrich, St. Louis, MO, USA) 10 mg/kg once per day by oral gavage prior to CIN induction. The dose of VAR was selected based on previous doses reported to be efficacious in rat studies [35]. The 5th group received AVA (AVA group; ≥98% (HPLC)) (Sigma-Aldrich, St. Louis, MO, USA) 50 mg/kg once per day by oral gavage prior to CIN induction. The dose of AVA was selected based on previous doses reported to be efficacious in rat studies [36]. NAC, VAR and AVA were dissolved in corn oil before administration. The G Power 3.1.9 software was used to calculate the number of rats [37]. The analysis was performed using the one-way analysis of variance (ANOVA). The value of the significance level was set at 0.05, and the power value was 0.80. With parameters in type I and II errors (5% and 20%) and using 4 administration groups, the number of rats was 5 per group.

**Figure 2:**
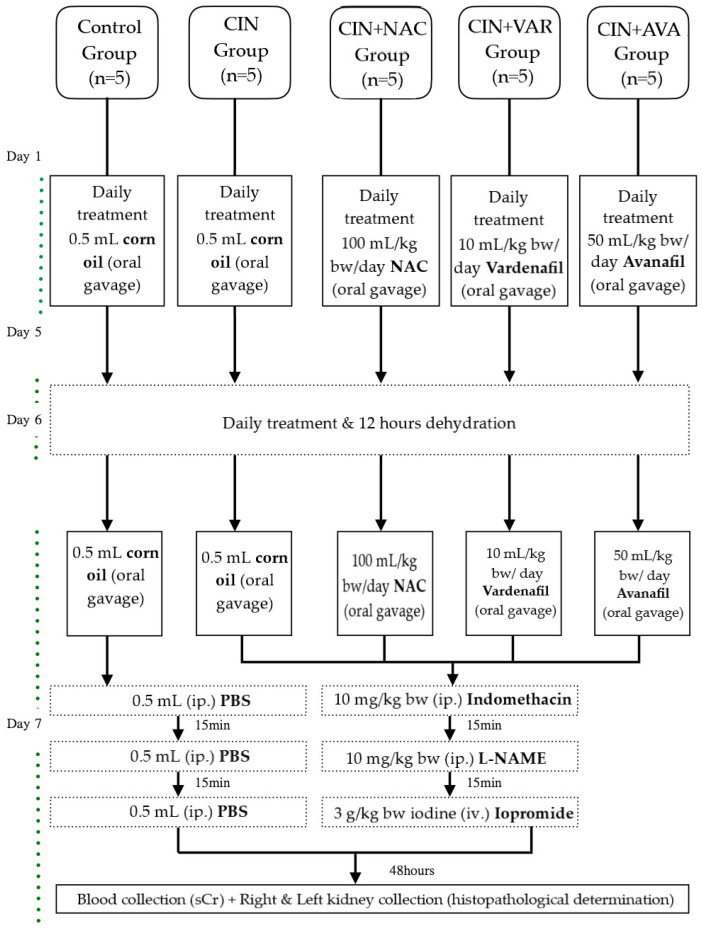
Experimental design.

CIN was induced as per our preceding protocols [21,22]. Briefly, on day 6, the animals were prevented from water consumption for 12 h in order to induce dehydration. On day 7, following the daily oral gavage, the animals from CIN, CIN+VAR, CIN+AVA, and CIN+NAC groups were injected intraperitoneally with an inhibitor of prostaglandin synthesis (indomethacin (Sigma-Aldrich, USA); 10 mg/kg bw) dissolved in phosphate buffered saline (PBS). After 15 min, the experimental groups received an inhibitor of nitric oxide (NO) synthesis (L-NAME (Sigma-Aldrich, USA); 10 mg/kg bw) dissolved in PBS intraperitoneally. After another 15 min, the experimental groups received iopromide (Ultravist 300 mg iodine per mL, Bayer Pharma, AG, Leverkusen, Germany, 3 g/kg bw iodine), injected in the tail vein within 10 min under light 3% sevoflurane anesthesia (Sevorane, Abbvie Deutschland GmbH & Co. KG, Wiesbaden, Germany). The control group received an identical quantity of PBS, following each stage of the CIN induction experimental process. At the beginning of the treatment and prior to sacrification, animals were weighed. All reagents used and the study experimental designs are summarized in Figure 2.

### 2.3. Biochemical Analyses

Blood was collected at 3 time points: immediately after CM exposure, 24 and 48 h after CM exposure (before sacrification). During the blood collection process, the rats were restrained in a special cage, and the sample was collected from the tail vein in 250 μL serum separation tubes (Arkay, Kyoto, Japan) using a 23 G needle. Serum creatinine (sCr), was determined using SPOTCHEM EZ SP-4430 Automated Veterinary Analyzer (Arkray, Japan). CIN was defined as an increase in sCr levels ≥25% at 24–72 h, following CM exposure compared to baseline levels.

### 2.4. Organ Collection and Preparation of Kidney Samples for ELISA Determinations

At 48 h after CIN induction, the animals were anesthetized using a mixture of ketamine (Alfamine 10%, Alfasan Int., Woerden, The Netherlands; 9.1 mg/kg bw) and xylazine (Alfazyne 2%, Alfasan Int., Woerden, The Netherlands; 9.1 mg/kg bw) and then sacrificed by exsanguination from abdominal aorta. Both kidneys were collected, half were fixed in formalin for histopathological evaluation, and the other half were weighted and homogenized on ice in a proportion of 100 mg tissue to 1 mL PBS and then kept at −20 °C overnight. Two freeze-thaw cycles were performed in order to break cell membranes, and finally, the homogenates were centrifuged at 2–5 °C, 5000× *g* for 5 min. The supernatant was deep frozen until further analysis. More precisely, the samples were deep frozen at −70 °C, and the supernatant was stored for 3 months before analysis. The samples were worked immediately after freeze-thaw, so only a freeze-thaw step was performed for the supernatant.

### 2.5. Determination of MMP-9 (Matrix Metalloproteinase 9/Gelatinase B), MMP-2 (Matrix Metalloproteinase 2/Gelatinase A), KIM-1 (Kidney Injury Molecule 1) and Cys-C (Cystatin-C) in Kidney Homogenates by ELISA

The levels of MMP-2, KIM-1 and Cys-C in kidney homogenates were assessed using the MMP-2 ELISA kit (catalog no. CSB-E07411r, Cusabio, Wuhan, China), KIM-1 ELISA kit (catalog no. CSB-E08808r, Cusabio, Wuhan, China), Cys-C ELISA kit (catalog no. CSB-E08385r, Cusabio, Wuhan, China) according to manufacturer instructions. The levels of MMP-9 in kidney were assessed using the MMP9 ELISA kit (catalog no. ABIN6730943, Antibodies-Online GmbH, Aachen, Germany). The limits of detection for the ELISA kits used were as follows: 0.625 ng/mL for MMP-2, 0.313 ng/mL for Kim-1, 7.8 ng/mL for Cys-C and 4.7 ng/mL for MMP-9.

### 2.6. Kidney Histopathological Evaluation

Half of the kidneys were collected and kept for 24 h in 4% paraformaldehyde. Then they were dehydrated in increasing concentration of ethanol solutions for 1 h in 70% solution, 1 h in 90% solution, and 5 h in 100% solution. Then the organs were fixed for 2 h in xylene and embedded in paraffin. Blocks were cut into slices of 25 µm with a microtome and stained with hematoxylin/eosin [21]. The histological evaluation was performed using a light microscope (Panthera L; Motic Europe, S.L.U, Wetzlar, Germany).

### 2.7. Data Analysis

Statistical analyses were performed using SPSS software (IBM Corp. Released 2021. IBM SPSS Statistics for Windows, Version 28.0. IBM Corp., Armonk, NY, USA). Groups were compared using one-way ANOVA, following normality check with Shapiro–Wilk test. Post-hoc comparisons were performed using Tukey test. Kruskal–Wallis test was used as appropriate. *p*-Value ≤ 0.05 was considered significant.

### 2.8. Ethical Statement

The experimental protocol followed all ethical principles according to the Declaration of Helsinki and was approved by the Ethical Committee of the University of Medicine and Pharmacy of Craiova, Romania (Pr. No: 32/22-05-2020).

## 3. Results

### 3.1. Biochemical Evaluation

**Figure 3:**
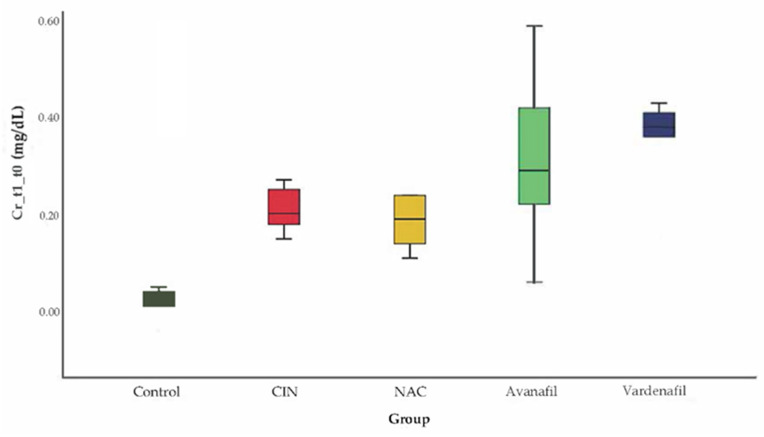
Variation of sCr levels at 24 h after CM exposure, no statistically significant difference among groups (ANOVA; *n* = 5 animals per group).

**Figure 4:**
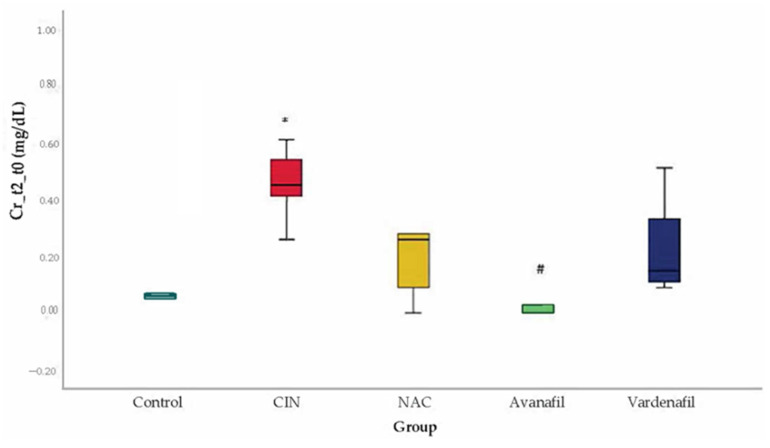
Variation of sCr levels at 48 h after CM exposure, *: statistically significant difference in CIN group compared to control, ^#^: statistically significant difference in CIN+AVA group compared to CIN (ANOVA; *n* = 5 animals per group).

CIN was induced successfully in all animals/groups at 24 h after CM exposure, since sCr levels increased over 25% compared to baseline (Figure 3). At 48 h after CM exposure, sCr levels exhibited a reducing pattern in all treatment groups compared to CIN group, albeit this reduction was statistically significant only in CIN+AVA group compared to CIN group (95% CI: −0.79, −0.10 *p* < 0.001) (Figure 4).

### 3.2. MMP-9 Evaluation

**Figure 5:**
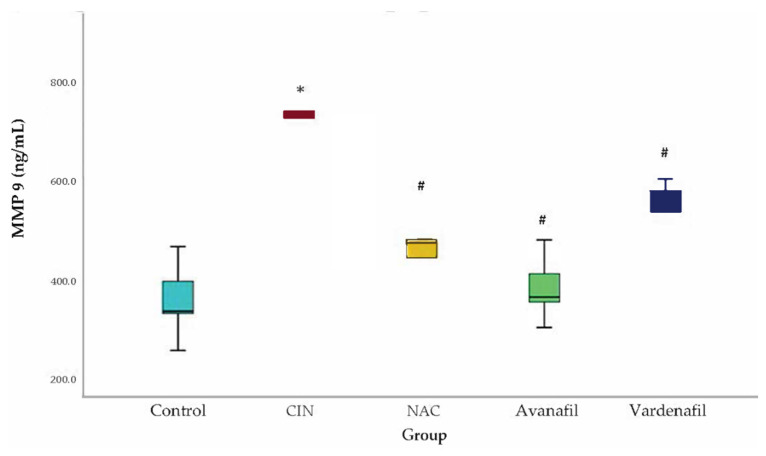
MMP-9 levels. Comparison of treatment groups with control and CIN at 48 h after CM exposure (at sacrification). *: statistically significant difference in CIN group compared to control, **^#^**: statistically significant difference in all treatment groups compared to CIN (ANOVA; *n* = 5 animals per group).

MMP-9 levels increased in all groups compared to the control with a statistical significance detected only for CIN group (95% CI: 200.80, 568.56 *p* < 0.001). In CIN group, MMP-9 levels were upregulated compared to CIN+NAC; CIN+VAR; and CIN+AVA groups, reaching statistical significance (95% CI: 113.36, 481.13 *p* < 0.001; 95%CI: 43.97, 411.73 *p* = 0.01; 95% CI: 174.63, 542.39 *p* < 0.001, respectively) (Figure 5).

### 3.3. MMP-2 Evaluation

**Figure 6:**
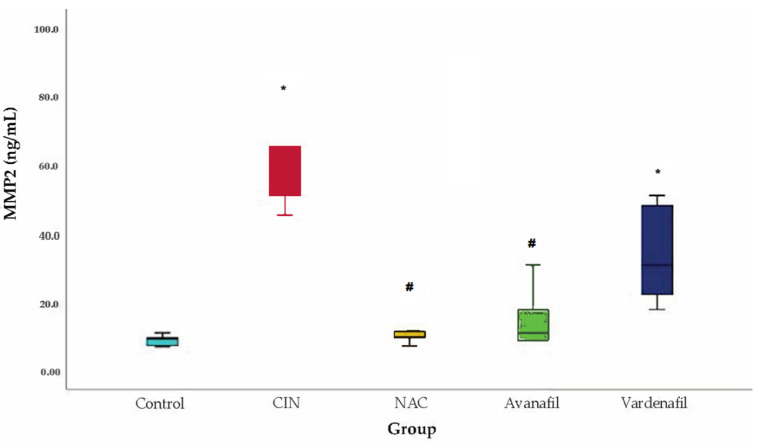
MMP-2 levels. Comparison of treatment groups with control and CIN at 48 h after CM exposure (at sacrification), *: statistically significant difference in CIN, CIN+VAR groups compared to control, ^#^: statistically significant difference in CIN+NAC, CIN+AVA groups compared to CIN (ANOVA; *n* = 5 animals per group).

MMP-2 levels increased in all groups compared to control, with statistically significant difference for CIN (*p* < 0.001) and CIN+VAR (*p* = 0.009) groups. Similarly, a decline in the MMP-2 levels was noted in all treatment groups compared to CIN group but only in CIN+NAC (*p* = 0.004) and CIN+AVA (*p* = 0.008) groups appeared to be statistically significant (Figure 6).

### 3.4. KIM-1 Evaluation

**Figure 7:**
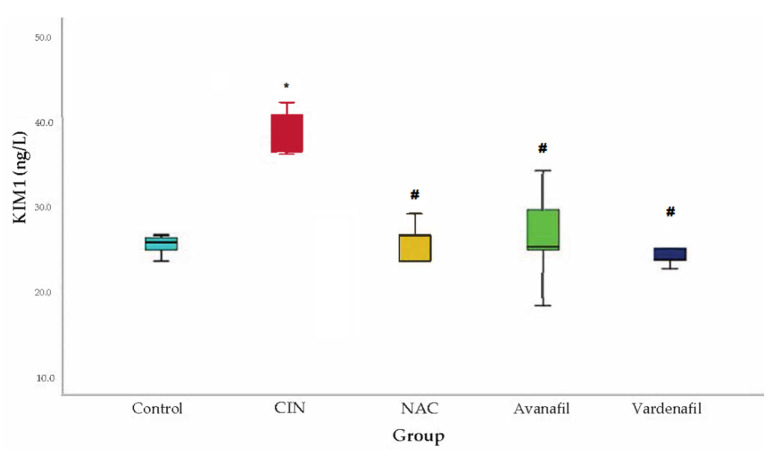
KIM-1 levels. Comparison of treatment groups with control and CIN at 48 h after CM exposure (at sacrification), *: statistically significant difference in CIN group compared to control, ^#^: statistically significant difference in all treatment groups compared to CIN (ANOVA; *n* = 5 animals per group).

In CIN group, KIM-1 levels demonstrated a significant rise compared to the control group. A statistically significant decline in KIM-1 levels was evident in all treatment groups compared to CIN group: CIN+NAC: 95% CI (6.77–23.22 *p* < 0.001), CIN+AVA: 95% CI: 3.91, 20.37 *p* < 0.002), CIN+VAR: 95% CI: (5.60, 22.05, *p* < 0.001) (Figure 7).

### 3.5. Cystatin-C Evaluation

**Figure 8:**
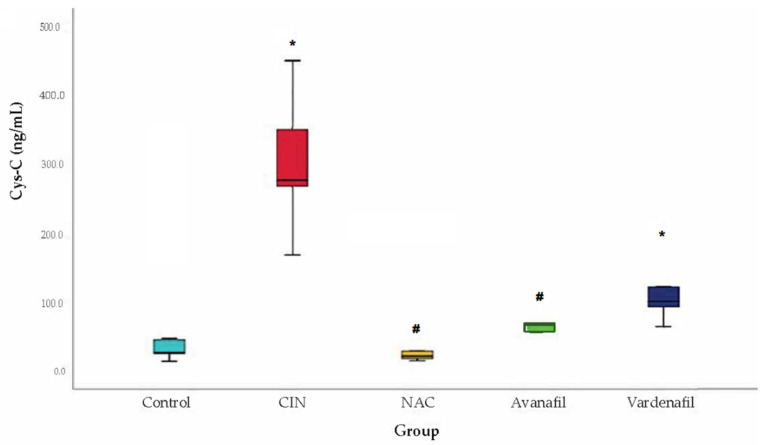
Cystatin-C levels. Comparison of treatment groups with control and CIN at 48 h after CM exposure (at sacrification), *: statistically significant difference in CIN, CIN+VAR groups compared to control, ^#^: statistically significant difference in CIN+NAC, CIN+AVA groups compared to CIN (ANOVA; *n* = 5 animals per group).

Cys-C levels increased significantly in CIN group compared to the control group. Regarding the treatment groups, Cys-C levels increased too compared to control, but the difference reached statistical significance only for CIN+VAR (*p* = 0.02) group. Cys-C levels increased in CIN group compared to CIN+NAC, CIN+VAR and CIN+AVA groups, however the difference was significant only for CIN+NAC (*p* < 0.001) and CIN+AVA (*p* = 0.02) groups (Figure 8).

### 3.6. Histopathological Evaluation

**Figure 9:**
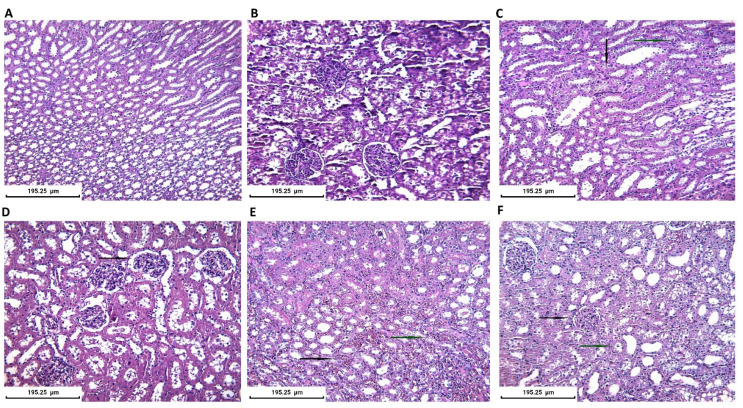
Histopathological kidney evaluation in all groups (Staining H&E): (**A**) Control group. Normal kidney aspect-tubular/medullar level. ×200; (**B**) Control group. Normal kidney aspect-glomerular/cortex level (×200); (**C**) CIN group. Tubular injury with moderate degeneration with granular cytoplasm (black arrow) and hypertrophic or hyperchromic nuclei (blue arrow) (×200); (**D**) AVA group. Enlargement of subcapsular space (black arrow) and minimal tubular architectural distortion (×200); (**E**) VAR group. Minimal tubular injury with medullar hemorrhagic foci (black arrow) and hyperemia (blue arrow) (×200); (**F**) NAC group. Cortex with normal glomerular aspect (black arrow) and minimal medullar injury with vacuolar degeneration in some tubules (blue arrow) (×200).

Half of the collected kidneys were fixed in formalin for histopathological evaluation. All histopathological evaluations were performed blindly and independently by two experts and subsequently the histopathological aspects were corroborated. The control group presented a normal microscopic appearance of the kidney. In the CIN group, medullar injuries were found with twisted tubes with distorted architecture, moderate granular degeneration, hypertrophic nuclei, hyperchromes, and discreet blood infiltration. For the CIN+AVA group the lesions were mostly situated in the renal cortex, and we described glomeruli with enlarged subcapsular space and some minimal medullar pathological findings regarding tubes with granular degeneration. CIN+VAR/CIN+NAC groups showed minimal changes in tubular architecture. All treatment groups presented close to normal kidney appearance. The histopathological results are presented in Figure 9.

## 4. Discussion

As previously reported, PDE5Is appear to have a renoprotective potential in AKI independently of its etiology and the agent applied, by probably influencing regional hemodynamics; promoting cell expression; and amplifying the mitochondrial response to oxidative stress [28]. To date, only sildenafil and tadalafil have been evaluated in this respect specifically in CIN animal models showing renoprotective properties [28]. The present study provides emerging evidence for the renoprotective effects of VAR and AVA in CIN rat model for the first time. This model was reproduced according to a previous study investigating the effect of NO and prostanoids on the sensitive to hypoxia renal medulla following CM administration [38]. CIN was introduced with dehydration followed by L-NAME/indomethacin administration and iopromide exposure. CIN was induced successfully in all the animals of the CIN group since sCr level was elevated ≥25% compared to baseline in all group members. In the VAR and AVA groups, the increase in sCr levels was limited at 48 h and were close to NAC and control groups.

Within the limited available literature regarding the potential protective/preventive effect of PDE5Is, there are reports for several biomarkers used and histological findings that were affected following the exposure to the iodinated CM [28]. The functional parameter sCr was evaluated and found to be raised in all studies, following CM administration [28]. sCr is the hallmark biomarker that has been utilized for years in order to reflect alterations in renal function, but its kinetics make it a late and non-precise molecule [3]. Emerging tissue, serum, and urinary biomarkers are eagerly studied in order to yield sensitive and accurate detection, treatment optimization, deflection of unnecessary interventions and avoidance of detrimental consequences associated with CIN [5,39]. In the present study, we focused on the effect of VAR and AVA on sCr, renal histopathology and four tissue biomarkers were sorted out from the array of analytes associated with CIN, after systematically searching the available literature [39], merely on the basis of their reported validity (KIM-1, Cys-C), novelty (MMP-2 and MMP-9) and the resources available for the present experiment.

Both sildenafil and tadalafil have been reported to lower sCr levels, demonstrating a positive effect against CIN [17,21,40,41,42]. Evidence from previous studies has indicated that KIM-1 has shown a renoprotective effect mainly in AKI, measured in urine or in renal tissue [28]. Although most of the studies have examined KIM-1 levels in the urine as an AKI indicator, it has been demonstrated that urinary KIM-1 levels reflect tissue KIM-1 and are associated with tubular damage of the kidney [43]. Additionally, several studies have shown that inhibition of MMP-2 and MMP-9 protects against AKI, as they are increased in the acute phase of kidney injury and modulate tubular/microvascular damage. However, the role of MMPs remains uncertain because there are knockout mouse studies showing that MMP-2 and MMP-9 may have preinjury effects [44]. Furthermore, Cys-C has been established as a reliable biomarker for early detection of AKI, hence it has been evaluated in CIN experiments. Özbek et al. demonstrated that CIN raised serum Cys-C levels but the administration of tadalafil immediately after CM reversed that effect to almost baseline values [40]. Along with oxidative stress, other potential pathophysiological mechanisms for CIN are renal vasoconstriction and vascular endothelial damage [3]. Almeida et al., apart from functional and oxidative changes, evaluated hemodynamic parameters such as renal blood flow and renal vascular resistance showing that diminished renal blood flow/elevated renal vascular resistance improved significantly following sildenafil administration [42].

Finally, there is evidence that CM induces significant histological alterations at the organ/tissue level. Histological alterations include increased organ weight and Bowman space, lobulated glomerulus, alterations of the macula densa region [22], medullary congestion [40], inflammatory cell infiltration, intratubular cast formation and obstruction, tubular vacuolization/degeneration-necrosis [17,41], and hemorrhagic changes within the kidney [22]. Most of the available evidence sources denote an attenuation of the effect of CM following PDE5I administration [28]. Both sildenafil and tadalafil induced improvement of histological findings close to baseline histology. Only two studies failed to show significant recovery of the CM-induced tissue damage but did not indicate any worsening effect [40,42]. In our study, the control group presented the normal appearance of the kidney, while twisted tubes with distorted architecture and granular degeneration were revealed in the CIN group, while all treatment groups presented close to normal kidney appearance, a finding indicating that all medications provided protection to the renal tissue against the deleterious effects of CM. More precisely, CIN+VAR/CIN+NAC groups showed minimal changes in tubular architecture, while CIN+AVA group showed glomeruli with enlarged subcapsular space but less intense compared to CIN group and some glomeruli with minimal granular degeneration.

KIM-1 is a transmembrane protein expressed by damaged renal tubular epithelial cells. KIM-1, also known as T-cell immunoglobulin and mucin-containing molecule (Tim-1), is transported to the urine by a metalloproteinase. KIM-1 function remains unclear, but it may be used as a biomarker of renal tubular injury. Under normal circumstances, KIM-1 is undetectable in urine and healthy kidney tissue. However, following ischemic or toxic injury, KIM-1 is highly expressed by the epithelial cells of proximal convoluted tubules [45]. Following AKI, KIM-1 mRNA rises, and its end-product protein is localized in the most affected region of the proximal tubules [46]. In the ischemic/reperfusion model, KIM-1 expression was reported to be dramatically increased [47]. In another study on rats with acute nephrotoxicity after exposure to various toxins (*S*-(1,1,2,2-tetrafluoroethylene)-l-cysteine, folic acid, and cisplatin), upregulation of KIM-1 expression/presence in the urine in response to exposure suggested that this protein may serve as a general biomarker for tubular injury/repair processes [48]. In humans with acute tubular necrosis [49] and various other renal diseases [43], KIM-1 expression was reported to be upregulated and associated with tubular/interstitial damage and inflammation. In renal tissue of humans with diabetic nephropathy, KIM-1 expression was reported to be associated with nephropathy progression and loss of kidney function [50]. In our study, we showed that pretreatment with VAR or AVA decreased the expression of KIM-1 in renal tissue compared to CIN group.

MMPs belong to a wide family of zinc-dependent proteinases (endopeptidases) that play a significant role in the remodeling of extracellular matrix (ECM) [51]. Most of them are expressed in the kidney, affecting cell proliferation, death, and angiogenesis [44]. MMP-2 and MMP-9 are mainly located in the renal glomerulus, interact with the endothelium, glomerulus, the epithelial cells promoting ECM alterations, and are linked to a number of renal pathophysiologies, both acute and chronic [52]. The degree and duration of the elevation of MMPs has been shown to reflect the degree of renal damage [53]. A study on CIN murine model concluded that increased MMP-9 correlates with apoptosis and worse prognosis [54]. In a cisplatin-induced AKI mouse model, pretreatment with doxycycline inhibited MMP-2 and MMP-9 activity in renal tissue indicating a renoprotective effect [55]. Nebivolol attenuated kidney damage, reducing the expression and level of MMP-2 and MMP-9, as indicated in a rat model of ischemia-reperfusion injury [56]. However, in mice with ischemia-reperfusion kidney damage and MMP-9 gene deletion, renal function was not preserved [57]. Accommodating for the fact that MMPs result in significant renal microvascular alterations, which affect the regional vascular and cellular properties, complete inhibition of these two peptidases might provoke the completely opposite unfavorable outcome such as aggravation of the deleterious effect of renal insult [44,52,56]. Apprehending the aforementioned, we have shown that VAR or AVA pretreatment results in decreased MMP-2 and MMP-9 expression in renal tissue.

Cys-C (cystatin 3) is produced by nucleated cells and is fully catabolized at proximal tubules [58]. Kidneys are the only organs that clear Cys-C and its serum level depends only on glomerular filtration rate (GFR). Therefore, any change in serum Cys-C levels reflects GFR change [59]. Cys-C constitutes a well-studied renal function marker in various forms of AKI, including CIN. A meta-analysis reported that serum Cys-C level is more sensitive than sCr for early diagnosis of AKI [60]. Another study on patients with CIN secondary to coronary angiography concluded that serum Cys-C levels peak at 24 h after CM exposure and that a 15% increase in serum Cys-C level represents a diagnostic cut-off [61]. Our study is the first to assess Cys-C levels in kidney tissue showing that both VAR and AVA decrease its expression compared to CIN group.

VAR has been evaluated in a very limited number of AKI animal studies only and never in a CIN model to date, while AVA has not been evaluated in this respect before [28]. Previous results appear to be renoprotective with antioxidant and anti-inflammatory effects, showing improvement of histological changes (close to normal), reduction in tubular apoptosis/vacuolar degeneration and reduction in sCr levels [28]. In accordance with previous studies, our study provided emerging evidence that VAR exerts renoprotective effect, decreasing all biomarkers tested. Furthermore, the present results are encouraging for AVA too, which reduced serum and tissue biomarkers close to normal, constituting a promising treatment for preventing AKI after CM exposure. Both medications, including NAC, resulted in close to normal kidney appearance in treated rats, a finding indicating that all three medications provide protection for the renal tissue against the deleterious effects of CM. As for the other PDEIs, the renoprotective mechanisms of VAR and AVA remain obscure. Previously proposed mechanisms of counteracting the cascade of changes caused by renal injury (Figure 1) [28] need to be elucidated in future studies.

We acknowledge that our study may present some limitations such as the lack of inclusion of target molecule groups (NAC, VAR, and AVA without the nephropathy model) to investigate how these molecules per se can modulate the parameters under evaluation and to help understand the observed effect in the nephropathy model; the potential small size of groups that could explain, for example, non-statistically significant results due to type II error; the lack of serum Cys-C data; the lack of urine test data such as investigation of proteinuria; the lack of data on other tissue biomarkers such as neutrophil gelatinase-associated lipocalin; and the lack of immunohistology data. Consequently, the novel results presented should be further validated in future studies.

## 5. Conclusions

The present study evaluated for the first time the potential renoprotective effects of VAR and AVA in a CIN rat model. CIN increases MMPs, KIM-1, and Cys-C expression in rat renal tissue. Pre-treatment with VAR and AVA appears to ameliorate CIN. These results provide emerging evidence that VAR and AVA may have the potential to prevent CIN.

## Data Availability

The dataset presented in this study is available from the corresponding author upon reasonable request.

## Abbreviations

**AKI**: acute kidney injury; **Akt kinase**: protein kinase B or PKB; **ANOVA**: analysis of variance; **ATP**: *adenosine triphosphate*; **AVA**: avanafil; **Bax**: B-cell lymphoma protein 2-associated X; **Bcl-2**: B-cell lymphoma protein 2; **c-GMP**: cyclic guanosine monophosphate; **CD**: cluster of differentiation; **CI**: confidence interval; **CIN**: contrast-induced nephropathy; **CM**: contrast medium/media; **Cys-C**: cystatin-C; **e-NOS**: endothelial *nitric oxide synthase*; **ECM**: extracellular matrix; **ERK**: extracellular signal-regulated kinase; **GFR**: glomerular filtration rate; **HPLC**: high-performance liquid chromatography; **Kim-1**: kidney injury molecule 1; **MMP**: matrix metalloproteinase; **NAC**: *N*-acetyl cysteine; **NF-κB**: nuclear factor kappa-light-chain-enhancer of activated B cells; **NO**: nitric oxide; **NOS**: *nitric oxide synthase*; **PBS**: phosphate buffered saline; **PDE5i**: phosphodiesterase 5 inhibitor; **PI3K**: phosphoinositide 3-kinase; **ROS**: reactive oxygen species; **sCr**: serum creatinine; **Tim-1**: T-cell immunoglobulin and mucin-containing molecule; **VAR**: vardenafil; **↓**: reduced; **↑**: increased.
